# Multicenter comparison of transcatheter aortic valve implantation with the self-expanding ACURATE neo2 versus Evolut PRO transcatheter heart valves

**DOI:** 10.1007/s00392-023-02194-4

**Published:** 2023-04-28

**Authors:** Tobias Rheude, Costanza Pellegrini, Martin Landt, Sabine Bleiziffer, Alexander Wolf, Matthias Renker, Jonas Neuser, Oliver Dörr, Abdelhakim Allali, Tanja K. Rudolph, Jan Martin Wambach, Julian D. Widder, Parminder Singh, Dominik Berliner, Hector A. Alvarez-Covarrubias, Gert Richardt, Erion Xhepa, Won-Keun Kim, Michael Joner

**Affiliations:** 1 Klinik für Herz- und Kreislauferkrankungen, Deutsches Herzzentrum München, Technische Universität München, Lazarettstr. 36, 80636 Munich, Germany; 2 Heart Center, Segeberger Kliniken, Bad Segeberg, Germany; 3 Heart and Diabetes Center North Rhine-Westphalia, Bad Oeynhausen, Germany; 4 Department of Cardiology, Elisabeth Hospital Essen, Essen, Germany; 5 Department of Cardiology, Kerckhoff Heart Center, Bad Nauheim, Germany; 6 Department of Cardiology and Angiology, Hannover Medical School, Hannover, Germany; 7 Medical Clinic I, Department of Cardiology and Angiology, University of Giessen, Giessen, Germany; 8 Hospital de Cardiología, Centro Médico Nacional Siglo XXI, IMSS, Cd. de México, México; 9 DZHK (German Center for Cardiovascular Research), Partner Site Munich Heart Alliance, Munich, Germany

**Keywords:** Transcatheter aortic valve implantation, Transcatheter heart valves, Self-expanding, ACURATE Neo2, Evolut PRO

## Abstract

**Background:**

New-generation self-expanding transcatheter aortic heart valves (THV) were designed to overcome technical constraints of their preceding generations. We sought to compare the efficacy and safety of the self-expanding ACURATE neo2 (Neo2) versus Evolut PRO (PRO) devices.

**Methods:**

Seven hundred nine patients undergoing transfemoral transcatheter aortic valve implantation (TAVI) with either Neo2 (*n* = 496) or PRO (*n* = 213) were included. Propensity score matching (PSM) was performed to account for differences in baseline characteristics. In-hospital and 30-day clinical outcomes were evaluated according to Valve Academic Research Consortium-3 criteria.

**Results:**

Baseline characteristics were comparable between both groups after PSM (Neo2: *n* = 155, Evolut Pro: *n* = 155). Technical success rates were high in both groups (Neo2: 94.8% vs PRO: 97.4%; *p* = 0.239). Need for permanent pacemaker implantation was less frequent with Neo2 compared with PRO (7.5% vs 20.6%; *p* = 0.002), whereas major vascular complications were more frequent with Neo2 (Neo2: 11.6% vs PRO: 4.5%; *p* = 0.022). Intended valve performance at discharge was high in both groups without relevant differences among groups (Neo2: 97.4% vs. 95.3%; *p* = 0.328).

**Conclusions:**

Short-term outcomes after TAVI using latest-generation self-expanding THV were excellent, with overall low rates of adverse events. However, Neo2 was associated with lower pacemaker rates and reduced the prevalence of moderate–severe paravalvular leakage. Transprosthetic gradients after TAVI were higher with Neo2 compared with PRO.

**Graphical abstract:**

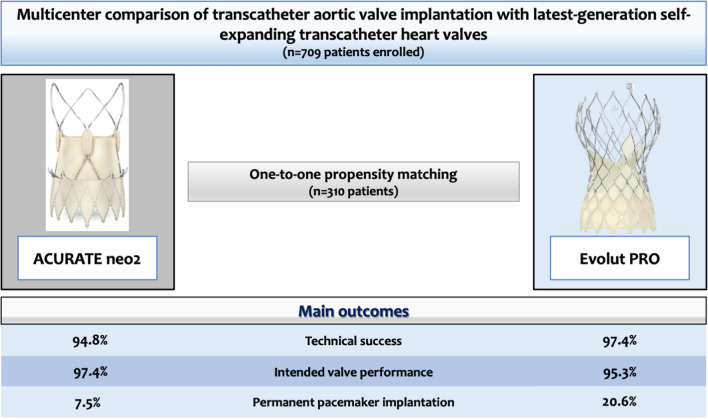

## Introduction

Transcatheter aortic valve implantation (TAVI) is an established treatment option for older patients with symptomatic severe aortic valve stenosis across the entire spectrum of surgical risk [1]. Refinement of procedural techniques and continuous device iteration played major roles in improving efficacy and safety of TAVI procedures, resulting in improved outcomes [2–4]. Recent studies showed excellent results even in younger patients at low surgical risk [5, 6].

Currently, both, self-expanding (SE) and balloon-expandable (BE) THV platforms are used on a global scale based on excellent results from several randomized trials and large multicenter registries over the last years, supporting their broad application [7, 8]. Among them, the SCOPE 2 randomized trial assigned patients with severe aortic valve stenosis to receive treatment with either ACURATE neo (*Neo*, Boston Scientific, Marlborough, MA, USA) or Evolut (Medtronic Inc., Minneapolis, MN, USA) SE-THV systems [7]. The Neo THV did not meet the prespecified non-inferiority criteria with regard to all-cause mortality or stroke at one year as compared to the Evolut THV platform and was further associated with elevated rates of moderate or severe aortic regurgitation at 30 days [7].

New iterations of both SE-THV platforms became recently available, the ACURATE neo2 (Neo2) and the Evolut PRO (PRO), to address limitations of earlier-generation devices, mainly paravalvular leakage (PVL) and need for permanent pacemaker implantation (PPI). Despite their broad application and very promising early results [9–11], comparative data are scarce.

Against this background, the purpose of this multicenter real-world study was to compare the performance of the latest-generation self-expanding Neo2 versus PRO THV systems.

## Methods

### Study population and procedures

**Fig. 1:**
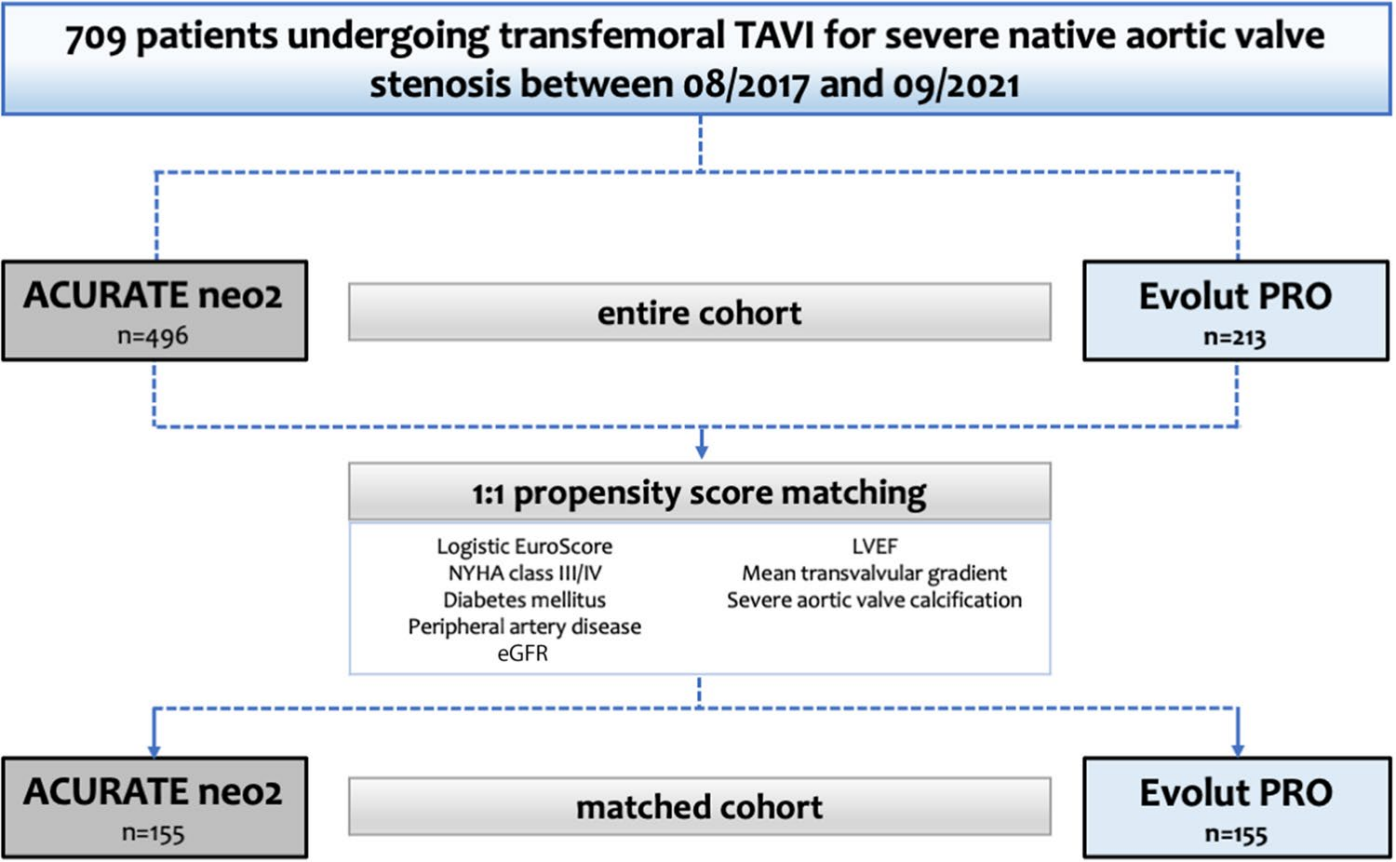
Study flow chart. LVEF left ventricular ejection fraction, NYHA New York Heart Association, TAVI transcatheter aortic valve implantation, eGFR estimated glomerular filtration rate

In this analysis, a total of 709 patients with symptomatic, severe native aortic valve stenosis undergoing transfemoral TAVI using Neo2 (*n* = 496) or PRO (*n* = 213) at seven centers in Germany (German Heart Center Munich; Heart Center, Segeberger Kliniken, Bad Segeberg; Heart and Diabetes Center North Rhine-Westphalia, Bad Oeynhausen; Kerckhoff Heart Center, Bad Nauheim; Hannover Medical School, Hannover; Department of Cardiology, Elisabeth Hospital Essen; Department of Cardiology and Angiology, University of Giessen) between August 2017 and September 2021 were retrospectively selected (Fig. 1). All patients were discussed by a local multidisciplinary heart team and found eligible for transfemoral TAVI. Patients underwent off-line analysis of multi-slice computed tomography (MSCT) using 3-mensio software (Pie Medical, Maastricht, Netherlands). Patients were treated in a hybrid operation theatre either in conscious sedation or general anesthesia. Procedures were performed according to international standards. Valve selection was left to the discretion of the operators performing the procedure. All patients provided written informed consent for the procedure and subsequent data collection per local practice. The study was approved by local ethics committees of the participating centers and complied with the Declaration of Helsinki.

### Device description

The THVs used in this study have been described in detail previously [10]. In brief, ACURATE Neo2 was granted Conformité Européenne (CE) mark in April 2020 based on the results of the Neo2 CE-mark study [10]. Like its predecessor, Neo2 is composed of a nitinol frame with axial, self-aligning stabilization arches and featuring supra-annular porcine pericardium leaflets. This new iteration was designed with a revised annular sealing technology designed to conform to irregular, calcified anatomies and an extended sealing skirt covering the entire waist of the stent, with the purpose to further reduce PVL. Moreover, a new radiopaque positioning marker was implemented to help navigate during valve positioning. Similar to its predecessor, the Neo2 valve is available in 3 sizes (small, medium and large), covering an annulus range from 21 to 27 mm.

The Evolut PRO THV is fabricated from porcine pericardial tissue, sutured in a supra-annular position into a self-expanding Nitinol frame. It adds an outer porcine pericardial wrap over the first 1.5 cells to enhance annular sealing and further reduce the incidence of PVL. It is repositionable and can be partially or fully recaptured until released from the delivery system to assist with optimal positioning [12]. The valve is available in 3 sizes (23, 26 and 29 mm), covering an annulus range from 18 to 26 mm.

### Definition of endpoints

Data were acquired during hospital stay and visits at the outpatient clinic, review of hospital records, contact of primary care physician, or by direct contact with the patient or her/his relatives at each center and collected in individual institutional databases. Data were consolidated in a joined database for statistical analyses. Collection involved demographic information, symptoms and co-morbidities, procedural data, as well as clinical and imaging assessment (echocardiography and computed tomography). Adverse clinical events were recorded throughout the follow-up period of 30 days after TAVI and procedural data, in-hospital complications and clinical endpoints were categorized according to Valve Academic Research Consortium (VARC)-3 criteria [13]. Transthoracic echocardiography was conducted after TAVI prior to discharge. PVL was graded based on transthoracic echocardiography according to a 3-class scheme as follows: none or trace, mild, moderate or severe. Aortic valve calcification was visually graded as mild, moderate or severe based on baseline computed tomography.

### Statistical analysis

Continuous variables are presented as mean with standard deviation or median with interquartile range and compared using Student’s *t* test or Mann–Whitney *U* test, as appropriate. Categorical variables are expressed as frequencies and proportions and compared using the Chi-square or Fisher’s exact test, as appropriate. Adverse events are reported as crude rates up to 30 days after TAVI. Event probabilities were compared for patients treated with Neo2 versus PRO using Cox proportional hazard regression analysis. Hazard ratios (HR) with their corresponding 95% confidence interval (CI) were computed.

To account for differences in baseline characteristics and the effect of a potential selection bias, propensity score matching (PSM) was performed using the R package *“MatchIt”* (Version 4.1.0, R Foundation for Statistical Computing, Vienna, Austria). A one-to-one nearest neighbor matching algorithm was used to identify one control case treated with Neo2 (*n* = 155) for each case treated with PRO (*n* = 155). Missing baseline data were imputed using the predictive mean matching function (R package *“Mice”*, version 3.13.0). Baseline, electrocardiographic and imaging characteristics (echocardiography or computed tomography) showing significant univariate differences between both groups or with known influence on outcome were included in the matching algorithm. These variables were: logistic European System for Cardiac Operative Risk Evaluation (Euro-)SCORE, New York Heart Association (NYHA) class III/IV, diabetes mellitus, peripheral artery disease, estimated glomerular filtration rate (eGFR), left ventricular ejection fraction (LVEF), mean transvalvular gradient and severe aortic valve calcification.

A 2-sided *p* value < 0.05 was considered statistically significant. Statistical analyses were performed using R (Version 4.1.3, R Foundation for Statistical Computing, Vienna, Austria) and IBM SPSS Statistics (Version 28.0 for Macintosh, IBM Corp., Armonk, NY, USA).

## Results

### Baseline patient characteristics

A total of 709 patients undergoing transfemoral TAVI with either self-expanding Neo2 (*n* = 496) or PRO (*n* = 213) THVs were included in this analysis. Baseline characteristics are displayed in Table 1. In the entire cohort, patients treated with Neo2 presented more frequently with NYHA class III/IV (68.5% vs. 60.6%; *p* = 0.046), had higher rates of diabetes (34.3% vs. 23.9%; *p* = 0.006) and peripheral artery disease (14.5% vs. 5.6%; *p* = 0.001), a better renal function (eGFR: 65 ml vs. 53 ml; *p* < 0.001), a better LVEF (60% vs. 55%; *p* < 0.001) and lower mean transvalvular gradients (42 mmHg vs. 47 mmHg; *p* < 0.001). Rates of severe aortic valve calcification were significantly higher with PRO compared with Neo2 (46.0% vs. 10.7%; *p* < 0.001), whereas bicuspid valves were equally distributed (Neo2: 3.6% vs. PRO: 2.8%; *p* = 0.658). A 1-to-1 propensity-score-matching analysis resulted in a total of 155 matched pairs (*n* = 310 patients in total). As shown in Table 1, there was no relevant difference in any baseline characteristic among both groups.

**Table 1:** Baseline characteristics

	Entire cohort (*n* = 709)			Matched cohort (*n* = 310)		
	Neo2 (*n* = 496)	PRO (*n* = 213)	*p* value	Neo2 (*n* = 155)	PRO (*n* = 155)	*p* value
Age (years)	82 [79–85]	82 [79–85]	0.395	82 [78–85]	82 [79–85]	0.677
Female gender	240 (48.4)	115 (54.0)	0.190	85 (54.8)	87 (56.1)	0.909
Body Mass Index (kg/m^2^)	26.5 [23.7–29.7]	26.5 [23.8–29.9]	0.827	25.7 [23.2–29.0]	26.5 [23.6–29.9]	0.127
Logistic EuroSCORE (%)	14.4 [8.1–23.1]	12.0 [8.0–21.2]	0.102	11.4 [6.7–20.3]	11.4 [8.0–20.0]	0.730
NYHA class III/IV	340 (68.5)	129 (60.6)	0.046	91 (58.7)	101 (65.2)	0.292
Arterial hypertension	432 (87.1)	189 (88.7)	0.620	128 (82.6)	137 (88.4)	0.197
Diabetes mellitus	170 (34.3)	51 (23.9)	0.006	37 (23.9)	39 (25.2)	0.895
Coronary artery disease	303 (61.1)	122 (57.3)	0.358	93 (60.0)	91 (58.7)	0.908
Previous percutaneous coronary intervention	172 (34.7)	73 (34.3)	0.932	52 (33.5)	50 (32.3)	0.904
Previous coronary artery bypass grafting	36 (7.3)	14 (6.6)	0.873	11 (7.1)	11 (7.1)	> 0.99
Previous myocardial infarction	44 (8.9)	16 (7.5)	0.659	12 (7.7)	10 (6.5)	0.826
Previous stroke	63 (12.7)	17 (8.0)	0.071	16 (10.3)	12 (7.7)	0.553
Chronic obstructive pulmonary disease	64 (12.9)	20 (9.4)	0.206	10 (6.5)	14 (9.0)	0.525
Peripheral artery disease	72 (14.5)	12 (5.6)	0.001	9 (5.8)	10 (6.5)	> 0.99
eGFR (ml/min/1.73m^2^)*	65 [48–84]	53 [39–71]	< 0.001	56 [43–75]	55 [42–74]	0.743
Previous pacemaker	58 (11.7)	26 (12.2)	0.899	21 (13.5)	24 (15.5)	0.747
Atrial fibrillation	203 (40.9)	79 (37.1)	0.358	69 (44.5)	54 (34.8)	0.104
Left bundle branch block	49 (9.9)	18 (8.6)	0.674	22 (14.2)	14 (9.0)	0.214
Right bundle branch block	52 (10.5)	19 (9.0)	0.681	17 (11.0)	13 (8.4)	0.565
Mean transvalvular gradient (mmHg)	42 [32–50]	47 [39–57]	< 0.001	46 [39–56]	45 [37–56]	0.924
Left ventricular ejection fraction (%)	60 [55–65]	55 [52–60]	< 0.001	60 [54–65]	56 [55–60]	0.055
Bicuspid valve	18 (3.6)	6 (2.8)	0.658	10 (6.5)	4 (2.6)	0.170
Mean aortic annulus diameter (mm)	23.6 [22.3–24.8]	24.0 [23.0–25.0]	0.555	23.6 [22.4–24.8]	23.8 [23.0–24.2]	0.748
Severe aortic valve calcification	53 (10.7)	98 (46.0)	< 0.001	47 (30.3)	51 (32.9)	0.714

Data are median [interquartile range] or *n* (%)

*eGFR* estimated glomerular filtration rate, *EuroSCORE* European System for Cardiac Operative Risk Evaluation, *NYHA* New York Heart Association

*Available in *n* = 655

### Procedural characteristics and clinical outcomes

Procedural characteristics and VARC-3 defined clinical outcomes of the matched cohort are depicted in Table 2. Pre-dilatation prior to valve implantation was more frequently performed with Neo2 compared with PRO (94.2% vs. 56.1%; *p* < 0.001). Moreover, contrast agent volume was significantly lower with Neo2 compared with PRO (40 ml vs. 97 ml; *p* < 0.001).

**Table 2:** Procedural characteristics and clinical outcomes of the matched cohort

	Neo2	Evolut PRO	*p* value
**Procedural characteristics**			
Valve size			
Small	31 (20.0)		
Medium	74 (47.7)		
Large	50 (32.3)		
23 mm		–	
26 mm		40 (25.8)	
29 mm		115 (74.2)	
Conscious sedation	154 (99.4)	115/117 (98.3)	0.579
Pre-dilatation	146 (94.2)	87 (56.1)	< 0.001
Post-dilatation	81 (52.3)	63 (40.6)	0.040
Procedural time (min)	48 [37–62]	53 [45–66]	0.003
Fluoroscopy time (min)	10.7 [8.1–14.2]	9.5 [7.1–13.1]	0.045
Contrast agent (ml)	40 [24–145]	97 [82–128]	< 0.001
**Clinical outcomes**			
Technical success (VARC-3)	147 (94.8)	151 (97.4)	0.239
Intended valve performance (VARC-3)	149 (97.4)	141 (95.3)	0.328
Device success (VARC-3)	142 (91.6)	142 (91.6)	> 0.99
Procedural mortality	0	0	–
Correct implant position	154 (99.4)	154 (99.4)	> 0.99
Second THV implanted	0	2 (1.3)	0.498
Annular rupture	0	0	–
Coronary obstruction	1 (0.6)	0	> 0.99
Conversion to open heart surgery	0	0	–
Major vascular complications	18 (11.6)	7 (4.5)	0.022
All stroke	3 (1.9)	6 (3.9)	0.501
New permanent pacemaker implantation*	10/134 (7.5)	27/131 (20.6)	0.002
Bleeding type 3/4 (VARC-3)	9 (5.8)	4 (2.6)	0.157
Myocardial infarction	0	0	–
Acute kidney injury stage 2/3	9 (5.8)	5 (3.2)	0.274
**Echocardiographic outcomes**			
Mean transvalvular gradient	9 [7–12]	8 [6–11]	0.001
Mean gradient ≥ 20 mmHg	3/153 (1.9)	0/148 (0)	0.055
Moderate to severe PVL	1/154 (0.6)	7/153 (4.6)	0.036

Data are median [interquartile range] or *n* (%)

*PCI* percutaneous coronary intervention, *PVL* paravalvular leakage, *THV* transcatheter heart valve, *VARC* Valve Academic Research Consortium

*Excluding patients with pacemaker at baseline

**Fig. 2:**
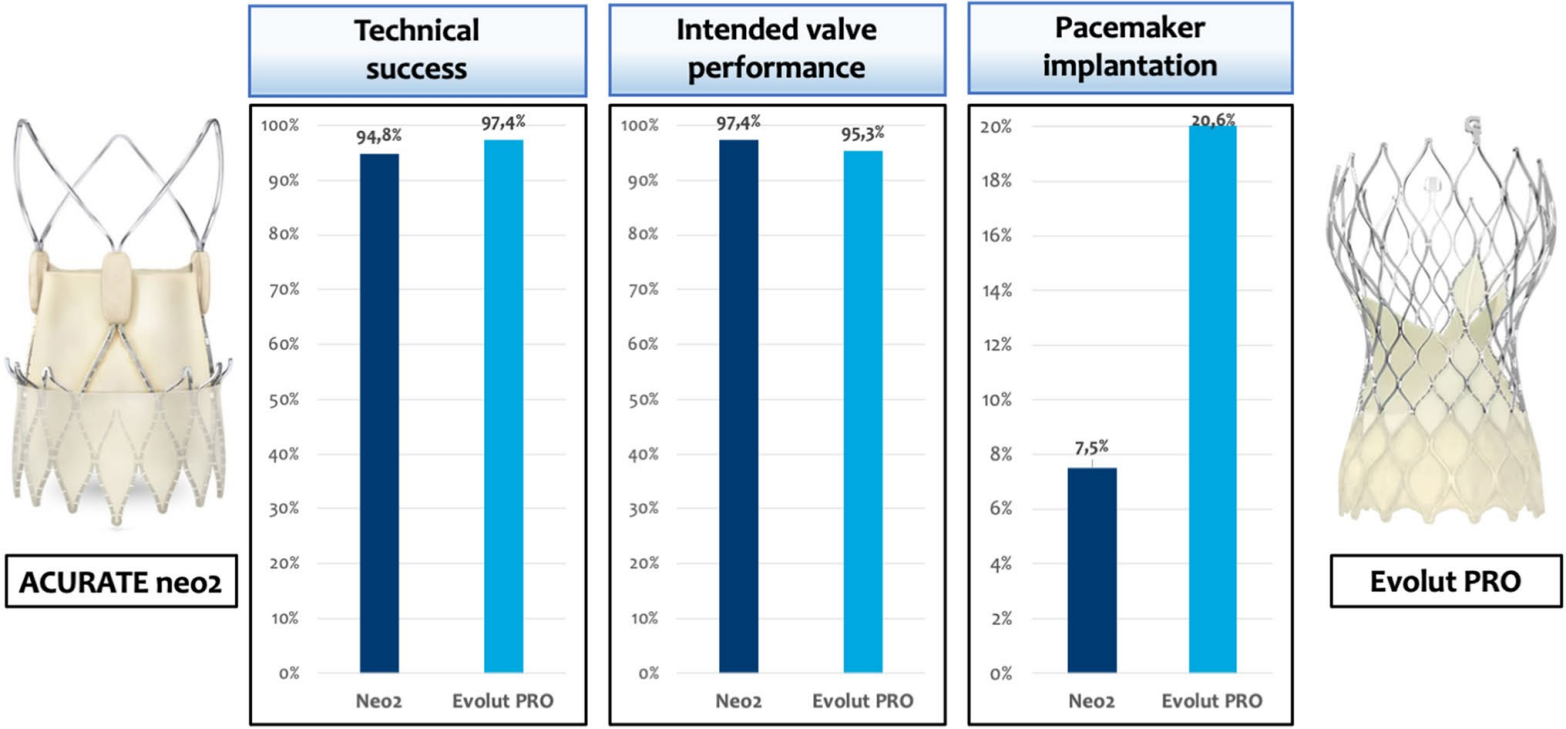
Major findings after transcatheter aortic valve implantation with ACURATE neo2 versus Evolut PRO

**Fig. 3:**
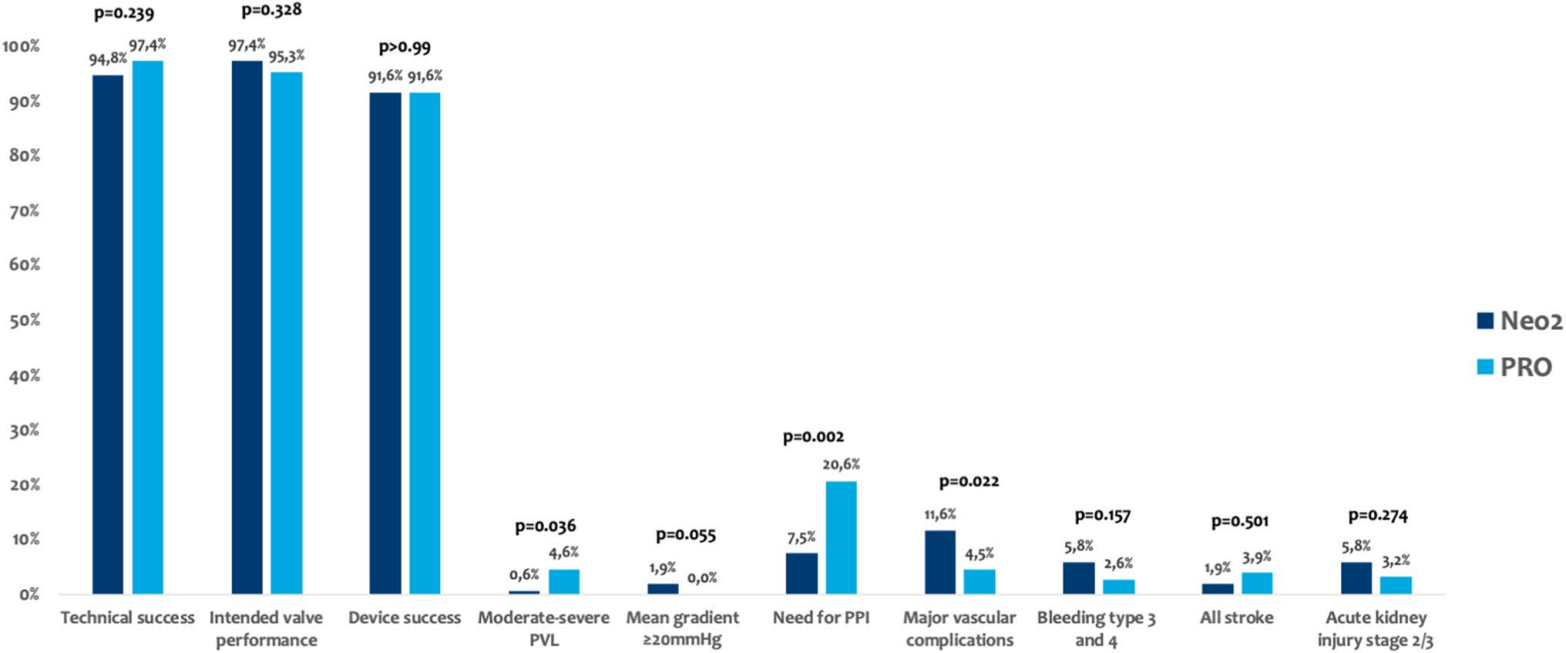
VARC-3 defined procedural complications and clinical outcomes. PVL paravalvular leakage, PPI permanent pacemaker implantation, VARC Valve Academic Research Consortium

Technical success rates were high in both groups (Neo2: 94.8% vs. PRO: 97.4%; *p* = 0.239; Figs. 2 and 3). Need for PPI was significantly lower in patients treated with Neo2 compared with PRO (7.5% vs. 20.6%; *p* = 0.002; Figs. 2 and 3). Third-degree atrioventricular block was the most frequent indication in 78% of cases (29/37). Of note, cusp overlap technique was only used in one-third of patients treated with PRO (48/153). Moreover, major vascular complications were more frequent with Neo2 (Neo2: 11.6% vs. PRO: 4.5%; *p* = 0.022). Rates of further peri-procedural complications were overall low and comparable with both devices (Fig. 3).

Intended valve performance at discharge was high in both groups without relevant differences (Neo2: 97.4% vs. 95.3%; *p* = 0.328; Figs. 2 and 3). Of note, rates of moderate–severe PVL were higher with PRO as compared with Neo2 (4.6% vs. 0.6%; *p* = 0.036). Consistently, rates of none or trace PVL were also higher with Neo2 compared with PRO (67.5% vs. 49.0%; *p* = 0.001). However, slightly increased mean transprosthetic gradients were found with Neo2 (Neo2: 9 [7–12] mmHg vs. PRO: 8 [6–11] mmHg; *p* = 0.001), with higher rates of elevated mean transprosthetic gradients ≥ 20 mmHg (Neo2: 1.9% (3/153) vs. PRO: 0% (0/148); *p* = 0.055).

The VARC-3 defined composite endpoint device success at 30 days was overall high in both THVs, without significant differences (Neo2: 91.6% vs. PRO: 91.6%; *p* > 0.99**; **Table 2).

## Discussion

This multicentric study compared new-generation self-expanding transcatheter heart valves for transcatheter aortic valve implantation. The main results can be summarized as follows: (1) Technical success rate was high with Neo2 and PRO THVs, (2) Permanent pacemaker rates after TAVI were higher after PRO implantation, whereas major vascular complications were more frequent with Neo2, (3) Intended valve performance at discharge was high with both THVs. In this regard, rates of moderate–severe PVL after TAVI were higher, whereas transprosthetic gradients were lower with PRO compared with Neo2.

Accumulating evidence with different THV platforms for the treatment of patients with severe aortic stenosis across the entire risk spectrum led to a continuous increase of TAVI procedures on a global scale. Widespread use of TAVI has been paralleled by technical refinement of available THVs aimed at improving procedural safety and efficacy [3, 4, 9, 11]. Self-expanding THVs with their deployment in a supra-annular position display several advantages, especially in certain subgroups of patients [14]; nevertheless elevated rates of significant PVL compared with balloon-expandable platforms belong to the drawbacks of earlier-generation SE-THVs [15, 16]. Both new iterations, Neo2 and PRO were designed with a revised annular sealing skirt aiming at further reduction of PVL rates after TAVI. Indeed, recent data from large multicenter registries demonstrated that improved annular sealing properties of the Neo2 were associated with a threefold lower frequency of moderate or greater PVL compared with the preceding Neo THV [3, 9]. Of note, the aforementioned advantage with regard to PVL was not counterbalanced by an elevated pacemaker rate, which was numerically even lower in patients treated with Neo2 [3, 9]. Likewise, technical refinements with Medtronic’s latest-generation THV, the Evolut PRO valve, resulted in very low rates of relevant PVL, while maintaining excellent hemodynamic performance as compared to the preceding generations in a large analysis with 18,874 patients from the Society of Cardiac Surgeons (STS)/American College of Cardiology (ACC) TVT Registry [17].

The VARC-3 defined composite endpoint technical success was high with both self-expanding THVs. The majority of peri-procedural and in-hospital complications were overall low and consistent with previous studies [3, 9, 11, 18], without relevant differences between the two treatment groups. However, the need for PPI after TAVI remains one of the major obstacles, even with current-generation devices, with a pooled incidence of 19% according to a recent meta-analysis [19, 20]. In our analysis, PPI rates after TAVI were significantly higher with PRO compared with Neo2. The 8% PPI rate with Neo2 in our study confirms recent data from other registries [3, 9]. The rate of new PPI after PRO implantation observed in our study is by trend higher as compared with previous studies ranging from 11.8 to 13.2% [18, 21]. Of note, continuous decrease of PPI rates was observed with device iterations of Medtronic’s THV platform with up to 38% in first-generation CoreValve [16], up to 20% in second-generation Evolut R [22] and up to 12% in latest-generation generation Evolut PRO in the Evolut PRO US Clinical Study [21]. This might not only be explained by technical refinements, but also by improved implantation techniques, such as the cusp overlap technique aimed at high valve implantation relative to the membranous septum in proximity to the non-coronary cusp [23]. This technique helped to minimize conduction disturbances and indeed resulted in significantly reduced PPI rates after TAVI when compared with the classical implantation technique [24, 25]. However, it was only used in one-third of patients treated with Evolut PRO in this multi-center all-comers study. Although pacemaker dependency rates among patients receiving PPI after TAVI were just around 33–36% at one year [26], adverse effects have been reported in these patients including reduced left ventricular function as well as an increased risk for heart failure hospitalizations and all-cause mortality at 1 year [27]. As even new left bundle branch block after TAVI has already been associated with an increased risk of all-cause death and heart failure hospitalization at 1-year follow-up in a large meta-analysis, further research is required to overcome this limitation of current-generation devices, especially in certain subgroups with an elevated risk for PPI after TAVI [27]. Rates of major vascular complications were more frequent in the Neo2-group. Previous studies investigating the Neo2 THV system reported lower rates of major vascular complication rates up to 6.0% [3, 9, 10]. Peripheral artery disease was significantly more common in the entire population and balanced between both groups after matching. As adverse events were site reported in this multicenter registry, individual data are not available to further illuminate this finding.

Intended valve performance at discharge was high with both devices, without relevant differences. As depicted in Fig. 3, rates of moderate or greater PVL after TAVI were lower with Neo2 compared to Evolut PRO. The low rates of moderate or greater PVL in the Neo2 group are in line with recent data from several international registries ranging from 0.6 to 3.5% [9–11, 28], with convincing data even in more challenging calcific anatomies [3]. This indicates that other risk factors beyond calcification might impact on residual PVL after successful THV implantation. The fact that technical refinements translate into better valve performance is of utmost clinical relevance as there is clear evidence that moderate or greater PVL adversely impacts on prognosis, including elevated rates of heart failure-related re-hospitalizations, valve re-interventions and mortality up to seven years after TAVI [29–31]. As the impact of mild PVL remains a matter of debate and might have detrimental effects in certain subgroups of patients [29, 32], and the given expansion to treat younger, lower-risk patients, post-procedural PVL should be eliminated completely to compete with surgical aortic valve replacement. In this regard, significantly more patients treated with Neo2 had none or trace PVL after TAVI. Post-procedural transprosthetic gradients were overall low with both self-expanding THV platforms, although mean transprosthetic gradients and rates of elevated transprosthetic gradients (≥ 20 mmHg) were lower after PRO implantation as compared to Neo2. Our results are consistent with previous studies investigating the hemodynamic performance of the preceding generation of the given SE-THV platforms with lowest gradients after implantation of Medtronic’s THV platform [33]. Bioprosthetic valve dysfunction with or without hemodynamic changes is complex with various underlying pathologies and mechanisms, as illustrated by updated VARC-3 criteria [13], and the impact of elevated post-procedural transprosthetic gradients with regard to clinical outcomes and valve durability remains a matter of debate and should be investigated in further studies [34–36].

## Limitations

This observational, multicenter study exhibits the inherent limitations of a retrospective, non-randomized study design. Severe aortic valve calcification was more frequent in the PRO-group of the entire population and well balanced between both groups after propensity score matching. Nevertheless, details regarding certain characteristics of calcification patterns, such as distribution of calcification (symmetric versus asymmetric, valvular versus LVOT calcification), degree of oversizing or implantation depth were not available and might have impacted on results, especially rates of residual PVL. Pre-procedural CT imaging data of the access vasculature regarding diameters of the ilio-femoral arteries, tortuosity and calcification were not available to further illuminate the different vascular complication rates. Moreover, the influence of additional unknown confounders cannot be excluded, despite rigorous matching algorithms. There was no core laboratory analysis of echocardiographic findings. A dedicated implantation approach (SLIM) was used at one center only and might have introduced bias with regard to significantly lower contrast amount in the Neo2-group, as it was predominantly used in Neo2 cases. Although clinical events were categorized according to standardized VARC-3 definitions, events were not adjudicated by an independent event adjudication committee. Event numbers were overall low; thus the results of this analysis should be interpreted with caution and should be confirmed in adequately powered prospective, randomized clinical trials.

## Conclusions

Short-term outcomes after TAVI with latest-generation self-expanding THVs were excellent with overall low rates of adverse events. Implantation of ACURATE neo2 was associated with lower pacemaker rates and reduced prevalence of moderate-severe paravalvular leakage. Evolut PRO showed lower transprosthetic gradients compared to ACURATE neo2.

## Abbreviations

CHF: Congestive heart failure
EuroSCORE: European System for Cardiac Operative Risk Evaluation
NYHA: New York Heart Association
PPI: Permanent pacemaker implantation
PSM: Propensity score matching
PVL: Paravalvular leakage
TAVI: Transcatheter aortic valve implantation
THV: Transcatheter heart valves
VARC: Valve Academic Research Consortium

## References

[1] Members WC, Otto CM, Nishimura RA (2021). 2020 ACC/AHA Guideline for the management of patients with valvular heart disease: executive summary: a report of the American College of Cardiology/American Heart Association Joint Committee on Clinical Practice Guidelines. J Am Coll Cardiol.

[2] Rheude T, Pellegrini C, Allali A et al (2022) Multicenter comparison of latest-generation balloon-expandable versus self-expanding transcatheter heart valves: Ultra versus Evolut. Int J Cardiol 357:115–120 10.1016/j.ijcard.2022.03.043 35337936

[3] Scotti A, Pagnesi M, Kim W-K (2022). Haemodynamic performance and clinical outcomes of transcatheter aortic valve replacement with the self-expanding ACURATE neo2. EuroIntervention.

[4] Rheude T, Pellegrini C, Lutz J (2020). Transcatheter aortic valve replacement with balloon-expandable valves: comparison of SAPIEN 3 ultra versus SAPIEN 3. JACC Cardiovasc Interv.

[5] Mack MJ, Leon MB, Thourani VH (2019). Transcatheter aortic-valve replacement with a balloon-expandable valve in low-risk patients. N Engl J Med.

[6] Popma JJ, Deeb GM, Yakubov SJ (2019). Transcatheter Aortic-Valve Replacement with a Self-Expanding Valve in Low-Risk Patients. N Engl J Med.

[7] Tamburino C, Bleiziffer S, Thiele H (2020). Comparison of self-expanding bioprostheses for transcatheter aortic valve replacement in patients with symptomatic severe aortic stenosis: SCOPE 2 randomized clinical trial. Circulation.

[8] Lanz J, Kim W-K, Walther T (2019). Safety and efficacy of a self-expanding versus a balloon-expandable bioprosthesis for transcatheter aortic valve replacement in patients with symptomatic severe aortic stenosis: a randomised non-inferiority trial. Lancet.

[9] Buono A, Gorla R, Ielasi A (2022). Transcatheter aortic valve replacement with self-expanding ACURATE neo2: postprocedural hemodynamic and short-term clinical outcomes. JACC Cardiovasc Interv.

[10] Möllmann H, Holzhey DM, Hilker M (2021). The ACURATE neo2 valve system for transcatheter aortic valve implantation: 30-day and 1-year outcomes. Clin Res Cardiol.

[11] Pellegrini C, Rheude T, Renker M (2022). ACURATE neo2 versus SAPIEN 3 Ultra for transcatheter aortic valve implantation. EuroIntervention.

[12] Manoharan G, Grube E, Van Mieghem NM (2020). Thirty-day clinical outcomes of the Evolut PRO self-expanding transcatheter aortic valve: the international FORWARD PRO study. EuroIntervention.

[13] Committee V-3 W, VARC-3 WRITING COMMITTEE, Généreux P (2021). Valve Academic Research Consortium 3: updated endpoint definitions for aortic valve clinical research. Eur Heart J.

[14] Voigtländer L, Kim W-K, Mauri V (2021). Transcatheter aortic valve implantation in patients with a small aortic annulus: performance of supra-, intra- and infra-annular transcatheter heart valves. Clin Res Cardiol.

[15] Husser O, Kim W-K, Pellegrini C (2017). Multicenter comparison of novel self-expanding versus balloon-expandable transcatheter heart valves. JACC Cardiovasc Interv.

[16] Abdel-Wahab M, Mehilli J, Frerker C (2014). Comparison of balloon-expandable vs self-expandable valves in patients undergoing transcatheter aortic valve replacement: the CHOICE randomized clinical trial. JAMA.

[17] Forrest JK, Kaple RK, Tang GHL (2020). Three generations of self-expanding transcatheter aortic valves: a report from the STS/ACC TVT Registry. JACC Cardiovasc Interv.

[18] Pagnesi M, Kim W-K, Conradi L (2019). Transcatheter aortic valve replacement with next-generation self-expanding devices: a multicenter, retrospective, propensity-matched comparison of Evolut PRO versus acurate neo transcatheter heart valves. JACC Cardiovasc Interv.

[19] Bruno F, D’Ascenzo F, Vaira MP (2021). Predictors of pacemaker implantation after transcatheter aortic valve implantation according to kind of prosthesis and risk profile: a systematic review and contemporary meta-analysis. Eur Heart J Qual Care Clin Outcomes.

[20] van Rosendael PJ, Delgado V, Bax JJ (2018). Pacemaker implantation rate after transcatheter aortic valve implantation with early and new-generation devices: a systematic review. Eur Heart J.

[21] Forrest JK, Mangi AA, Popma JJ (2018). Early outcomes with the Evolut PRO repositionable self-expanding transcatheter aortic valve with pericardial wrap. JACC Cardiovasc Interv.

[22] Grube E, Van Mieghem NM, Bleiziffer S (2017). Clinical outcomes with a repositionable self-expanding transcatheter aortic valve prosthesis: the International FORWARD Study. J Am Coll Cardiol.

[23] Tang GHL, Zaid S, Michev I (2018). “Cusp-Overlap” view simplifies fluoroscopy-guided implantation of self-expanding valve in transcatheter aortic valve replacement. JACC Cardiovasc Interv.

[24] Pascual I, Hernández-Vaquero D, Alperi A (2022). Permanent pacemaker reduction using cusp-overlapping projection in TAVR: a propensity score analysis. JACC Cardiovasc Interv.

[25] Aladham A, Gada H, Wang Y (2022). Incidence of permanent pacemaker implantation using the cusp overlap technique: a large single-center analysis. JACC Cardiovasc Interv.

[26] Costa G, Zappulla P, Barbanti M (2019). Pacemaker dependency after transcatheter aortic valve implantation: incidence, predictors and long-term outcomes. EuroIntervention.

[27] Faroux L, Chen S, Muntané-Carol G (2020). Clinical impact of conduction disturbances in transcatheter aortic valve replacement recipients: a systematic review and meta-analysis. Eur Heart J.

[28] Rück A, Kim W-K, Kawashima H (2021). Paravalvular aortic regurgitation severity assessed by quantitative aortography: ACURATE neo2 versus ACURATE neo transcatheter aortic valve implantation. J Clin Med.

[29] Chau K, Chau KC, Chen S (2022). Paravalvular regurgitation after transcatheter aortic valve replacement in intermediate-risk patients: a pooled PARTNER 2 study. EuroIntervention.

[30] Ludman PF, Moat N, de Belder MA (2015). Transcatheter aortic valve implantation in the United Kingdom: temporal trends, predictors of outcome, and 6-year follow-up: a report from the UK Transcatheter Aortic Valve Implantation (TAVI) Registry, 2007 to 2012. Circulation.

[31] Zahn R, Werner N, Gerckens U (2017). Five-year follow-up after transcatheter aortic valve implantation for symptomatic aortic stenosis. Heart.

[32] Okuno T, Tomii D, Heg D (2022). Five-year outcomes of mild paravalvular regurgitation after transcatheter aortic valve implantation. EuroIntervention.

[33] Regazzoli D, Chiarito M, Cannata F (2020). Transcatheter self-expandable valve implantation for aortic stenosis in small aortic annuli: the TAVI-SMALL Registry. JACC Cardiovasc Interv.

[34] Abdelghani M, Mankerious N, Allali A (2018). Bioprosthetic valve performance after transcatheter aortic valve replacement with self-expanding versus balloon-expandable valves in large versus small aortic valve annuli: insights from the CHOICE Trial and the CHOICE-Extend Registry. JACC Cardiovasc Interv.

[35] Rheude T, Pellegrini C, Cassese S (2020). Predictors of haemodynamic structural valve deterioration following transcatheter aortic valve implantation with latest-generation balloon-expandable valves. EuroIntervention.

[36] Rheude T, Pellegrini C, Stortecky S (2021). Meta-analysis of bioprosthetic valve thrombosis after transcatheter aortic valve implantation. Am J Cardiol.

